# 'Reaching the hard to reach' - lessons learned from the VCS (voluntary and community Sector). A qualitative study

**DOI:** 10.1186/1472-6963-10-92

**Published:** 2010-04-08

**Authors:** Sarah M Flanagan, Beverley Hancock

**Affiliations:** 1 Department or Primary Care Clinical Sciences, University of Birmingham Edgbaston, Birmingham, B15 2TT, UK

## Abstract

**Background:**

The notion 'hard to reach' is a contested and ambiguous term that is commonly used within the spheres of social care and health, especially in discourse around health and social inequalities. There is a need to address health inequalities and to engage in services the marginalized and socially excluded sectors of society.

**Methods:**

This paper describes a pilot study involving interviews with representatives from eight Voluntary and Community Sector (VCS) organisations. The purpose of the study was to explore the notion of 'hard to reach' and perceptions of the barriers and facilitators to accessing services for 'hard to reach' groups from a voluntary and community sector perspective.

**Results:**

The 'hard to reach' may include drug users, people living with HIV, people from sexual minority communities, asylum seekers, refugees, people from black and ethnic minority communities, and homeless people although defining the notion of the 'hard to reach' is not straight forward. It may be that certain groups resist engaging in treatment services and are deemed hard to reach by a particular service or from a societal stance. There are a number of potential barriers for people who may try and access services, including people having bad experiences in the past; location and opening times of services and how services are funded and managed. A number of areas of commonality are found in terms of how access to services for 'hard to reach' individuals and groups could be improved including: respectful treatment of service users, establishing trust with service users, offering service flexibility, partnership working with other organisations and harnessing service user involvement.

**Conclusions:**

If health services are to engage with groups that are deemed 'hard to reach' and marginalised from mainstream health services, the experiences and practices for engagement from within the VCS may serve as useful lessons for service improvement for statutory health services.

## Background

The notion 'hard to reach' is a contested and ambiguous term [1] that is commonly used within the spheres of social care and health, especially in discourse around health and social inequalities.

Reducing inequalities in health and health provision is a key theme underpinning the NHS Plan and National Service Framework' [2]. This same report acknowledged that certain groups are marginalized from services and therefore 'harder to reach' for health services whose goal is to provide appropriate and equitable health care for all populations. The Framework stated that primary care has a significant role to play in providing services that reach those in the greatest need but a Home Office report in 2004 [3] described the 'hard-to-reach' as 'inaccessible to most traditional and conventional methods for any reason.'. On the other hand, voluntary and community organisations (VCOs) are considered 'uniquely placed to reach marginalised groups' [4] and the 'hardest to reach' [5], and play an important role in the provision of health and social care services for these groups [6].

This paper describes the first stage of a planned study aimed at improving understanding of the 'hard to reach' and improving access to primary health services for 'hard to reach' groups. Acknowledgement of the established role of the Voluntary and Community Sector in providing services for these groups informed a decision to begin the study by exploring the perceptions and experiences of organisations from this sector.

This first stage of the study had two research objectives -

◦ To describe how service providers from the voluntary and community sector (VCS) conceive of the notion 'hard to reach'.

◦ To explore perceptions of the barriers and facilitators to accessing services for 'hard to reach' groups.

Using the search terms: 'hard to reach populations' 'hidden populations', 'marginalized populations', a literature search was conducted using the following databases: Books @ Ovid, Journal @ Ovid Full text, British Nursing Index (BNI), CAB Abstracts (1973-2006), CINAHL, EMBASE, Ovid Medline, PSYCINFO, Sociological Abstracts and Web of Science. Searches were restricted to English-language papers and limited from 1997 to present day. A search of the grey literature was also conducted using the Home Office and Department of Health websites. A Google search was undertaken limited to last 12 months using the term 'hard to reach Groups.'

Despite the apparent familiarity of the term and its use in social research and public policy, there would appear to be a lack of consensus about the meaning of the term. Within the literature, 'hard to reach' is often synonymised with other terms and the sheer multiplicity of alternatives reflects the divergence in the discourse as well as the difficulty in arriving at a definitive description of its meaning. 'Vulnerable', transient' [7] 'marginalised' 'refusers' [8], 'hidden' [9], 'forgotten populations' [10], 'underserved' [11], 'special populations' [12] 'disadvantaged populations' [13] are terms that have all been utilised in the literature pertaining to addressing issues faced by the 'hard to reach', or indeed those who are trying to reach them. There was an overarching sense that trying to engage the 'hard to reach' is problematic, as described almost two decades ago by Freimuth & Mettger [14](1990) and this may be due to the inherent ambiguity and lack of clarity of definition. Assignation of the term appears largely dependent upon the context of the organisation doing the reaching.

The 'hard to reach' groups most commonly identified in the literature are sex workers, drug users, people living with HIV and people from lesbian, gay, bisexual, transgender and intersex communities [15-17], but there are a number of other groups to which the description applies including asylum seekers, refugees, black and minority ethnic communities (BME), children and young people, disabled people, elderly people [18] and traveller families [19]. The 'hard to reach' may be people who are stigmatised due to the fact they are perceived as being somehow different.

If we think in terms of stigma and social exclusion in a healthcare context, the 'hard to reach' may include people with a variety of conditions or life limiting illnesses. This may include people with congenital abnormalities [20] or genetic conditions [21]; people with hearing loss [22]; people with mental health problems [23,24].

It is important to address also the notion that there are populations who are deemed 'hard to reach' but who are 'non-associative'; that is they do not generally associate with other members [25]. These populations are comparatively under-researched and it is acknowledged that trying to gain access to disparate and often isolated individuals, although labour intensive, is an important area of consideration for researchers and indeed service providers working with the 'hard to reach.'

'Hard to reach' audiences have been defined as 'inaccessible to most traditional and conventional methods for any reason' [3], highlighting the difficulty facing statutory health care provision. The voluntary and community sector has arguably been more successful in penetrating some of the barriers for the 'hard to reach' and has an important role to play in the understanding of service delivery provision.

There are around half a million voluntary and community organisations (VCOs) in the UK and there is a long tradition of voluntary action and community service in this country [4]. They have been used increasingly to deliver public services, especially over the last ten years [26-28].

The VCS is a term used to describe self-governing organisations working in areas of public benefit and they are 'value-driven' in that they 'exist for the good of the community' [29]. They can range in size from small community based groups or projects to large national and international organisations.

As the term suggests, these services often rely upon volunteers for service provision and delivery and are characterised by their independence from the 'formal structures of government and the profit sector'.

However, in practice it appears that the sector 'defies easy definition' [30] and it is suggested that the inherent ambiguity and variation in terms of how various organisations define and describe themselves is the very reason they are able to provide such diversity and flexibility.

### Research Objectives

The aim of the study was to explore 'hard to reach'-from the experiences and perspectives of the VCS. The findings would contribute to a larger study considering how these maybe translated to provision of services in primary health care.

The specific objectives of the work described in this paper were:

◦ To describe how service providers from the voluntary and community sector (VCS) conceive of the notion 'hard to reach'.

◦ To explore perceptions of the barriers and facilitators to accessing services for 'hard to reach' groups.

## Methods

This study utilised a qualitative design, since qualitative methods are well placed to explore sensitive and relatively poorly understood aspects of social life [31]. Semi-structured interviews were conducted with purposefully sampled team leaders or practitioners from VCS organisations across Birmingham.

Purposive sampling or criterion based sampling [32,33] was used to enable recruitment of participants who have particular experiences and roles that are pertinent to the study objectives.

The Birmingham Index of Voluntary Organisations was employed to access details of organisations working with 'hard to reach groups'. Letters of invitation were sent to 30 organisations and these were followed up by a telephone call, introducing the researcher and offering further details about the project. The organisations contacted included services working with people with mental health problems; homeless and poorly housed people; problem drug users, people identifying as gay, lesbian, bi-sexual and transgender; parents of young children in disadvantaged areas; people from BME communities; people working as carers; older adults; people with learning difficulties. These organisations were contacted as the groups they worked with are described in the literature pertaining to stigmatised, marginalised or hard to reach groups [23,34,15,17,34-38].

The initial plan was to interview service leads or managers as it was felt that they may be in the best position to give both an overview of their services, as well as providing accounts of front-line experience. However, it was not always possible to interview team leads, and participation was delegated by the service manager/team leads to other practitioners from within the projects, who in all but one case held a management role of some kind within the project. Eight services agreed to participate and the organisations worked with the following groups - young people, members of the Irish community, homeless people, people from BME communities living with HIV and people from the lesbian, gay and transgender communities, families and carers of drug users, young homeless people and people with mental health problems. The organisations that declined to participate cited time restrictions as their reason for non-participation.

The literature review on 'hard to reach groups' informed the development of the topic guide for the semi structured interviews. The topic guide covered the following areas:

• remit of your organisation and the client group you work.

• Experience of working with this group/in this field.

• Understanding of the term hard to reach.

• Who do you regard as 'hard to reach'?

• Why is it difficult to reach these groups? Why do you think they don't come?

• What measures have been put in place to engage the 'hard to reach'?

• Examples of successes in engaging the 'hard to reach'.

Ethical approval was sought and obtained from the university ethics committee. IRAS approval was not required as no NHS patients or employees were interviewed.

All interviews took place at the participants' place of work. All Participants agreed for the interviews to be audio-recorded and signed a consent form to reflect this.

Recorded interviews were transcribed by SF and each transcript was checked for any errors or omissions. Each manuscript was read and the emerging themes and key points were highlighted. Transcripts were analysed by both authors to ensure reliability of interpretation of the results. Labels and categories are used to 'organise and analyse the data' [32] by assigning key themes and emerging concepts to the raw data. This thematic approach to analysis provides a focus on the identifiable themes and patterns that emerge from the raw data.

Three broad areas emerged which include the following

◦ How the term 'hard to reach' is defined

◦ What barriers the 'hard to reach' face in accessing services.

◦ What can be done to engage 'the hard to reach'

Each of these broad themes comprised a number of categories which are discussed below. Quotations have been used throughout to add power to participants' accounts, and to encapsulate the views and experiences of individual participants [39].

## Results

### How the term 'hard to reach' is defined

Two respondents alluded to the apparent familiarity of the term 'hard to reach', for example, "one of those terms that people bandy about" while also indicating their unease with it. As one respondent explained (R5): "The problem I have with the term is that I don't really feel as if groups are actually hard to reach." before going on to suggest that it would be better to "find some other terminology to explain [it]." The term was attributed to describe the service (non) user and attributed to 'services' themselves.

### The unengaged - non users of services

There was an overall sense here that one conception of the 'hard to reach' refers to the people who are not seen in services. The "unengaged" was used to by one respondent to refer to those "who don't come to see us" (R1). Two other respondents described them as; "groups and communities who aren't accessing a particular service for whatever reason" (R5) and those who "traditionally wouldn't seek support from the usual avenues" (R6).

It is suggested that there are certain characteristics pertaining to how we may commonly define the notion of the 'hard to reach':

*People that would generally have difficulty accessing services... younger and more vulnerable people... you are talking about people who are more deprived, who are less well educated... may have restrictions placed upon them by society generally or their more narrow social group*. (R1)

This respondent further intimated that this does not tell the whole story and acknowledged in the context of providing a service for young people that the "white middle classes" may lead "controlled lives" that may inhibit them accessing particular services. This alternative explanation of the meaning of the term; the sense that the term 'hard to reach' can refer to those inhabiting a more privileged section of society [40,41] is an issue that is not raised in the remaining interviews but is worthy of note. More commonly we found that the responses support the sense that the 'hard to reach' are in some ways more needy; more marginalized; more likely to have experienced poverty, whether that be economic poverty or poverty of opportunity [7,19,9].

Lack of choice is another feature that was used to explain why a group maybe 'hard to reach' -

*You don't have those choices... either you can't afford them because you've got limited income and you haven't got the information either to be able to make an informed choice *(R7)

The transgender community is also particularly stigmatised [42]. The Diagnostic and Statistical Manual of Mental Disorders (DSM) includes 'Gender Identity Disorder' as a category and this 'continues to raise questions of consistency, validity, and fairness' [43]. To help overcome this, the project employs a transgender doctor to help engage with members of the transgender community.

### 'Hard to reach' understood from a service and societal stance

It is difficult to define the 'hard to reach' without thinking of the wider societal issues that contribute to or create such conditions. Service restrictions and limitations may mean that it is the services themselves that are 'hard to reach.'

One respondent alluded to the sense that it may be the way that an organisation is configured that creates this notion of the 'hard to reach' and she stated that the term can mean "people to whom we put up some barriers" -

*it makes it sound like the fault of the non service-user - "you are hard to reach" - like they are sat up on a shelf and we have got to lure them down with biscuits, or something.... actually if you just got a ladder and sat next to them that would be fine*. (R1)

Other ways of describing this were given in terms of 'hard to reach' individuals having "fallen through the net" (R7) or being "left out of the loop." This suggests a sense that certain groups or individuals are being excluded from services and this is the fault of the services themselves -

*organisations in the mainstream don't know ... how to reach them, how to engage them in activities, don't know where to find certain people*. (R2)

These people have in some way been let down by the system:

"for whatever circumstances [they] have been through the system and have either failed the system of the system has failed them." (R3)

One respondent made explicit the sense that the term is inappropriately attributed to groups and communities [18]. Instead, he stated that "it is about looking at our own services and looking at why individuals aren't engaging.

R6 suggested his organisation aims to engage

*the hardest to reach people who traditionally wouldn't seek support from the usual avenues and traditionally hadn't succeeded very well in either society or within the education system*.

Two key points are raised here; firstly of somebody being on the margins of what is considered to be the mainstream of society and secondly, a sense that the reason for this maybe due to a failure in the system.

This sentiment is developed and made more explicit later when the respondent intimates that the problem may be systemic -

*It's just about pushing them in that direction and perhaps that's where our school education doesn't quite serve everybody as well as it should*.

This respondent also stated that "we are very good at stereotyping and boxing people in" and he uses a quotation from a service user to demonstrate this notion:

*I am not "hard to reach", generally people don't know how to reach me*.

### Barriers and Facilitators to Services

In order to understand how appropriate services can be offered to the 'hard to reach', the experiences of VCS services providers in providing services to these groups were explored. Service providers were asked to describe the kinds of barriers they face in offering services to 'hard to reach' clients, as well as the factors that helped to facilitate the engagement of these groups.

### Barriers

#### Previous experiences of accessing services

A number of participants made explicit reference to the fact that many potential service users may not engage because of their previous experiences of accessing services. In particular, it would seem that statutory services were conceived as being particularly impenetrable [44], thus discouraging individuals to access help.

The sense that such experiences may begin in the formative years is indicated by one respondent who suggested that the "experience of the education system as being quite negative" (R6) is likely to have an impact upon future expectations.

#### Physical Factors

There are logistical factors to be considered that may serve as barriers to accessing services, including factors such as location and opening times.

One respondent indicated that "geography and transport" were barriers to services, but also the fact that young people may be conscious of gang boundaries:

quite a lot of young people aren't sure if it is ok for them to go to certain places. (R1)

A similar point was made by R7 who explained that their particular project moved location from the middle of an estate because:

*a lot of young people wouldn't use it, because it is all territorial*.

Another respondent (R7) suggested that geography may be a barrier to accessing services as potential service users "don't know we are here."

However, once people become aware of the location and are engaged in the service, limitations can arise in terms of how many people can be accommodated. By becoming victims of their own success, organisations may inadvertently exclude some more needy but less vocal members of their target audience. This idea is encapsulated by this quotation from R1;

*the worry is that when you are very busy... it's the more vulnerable that you squeeze out*.

#### Funding and Partnership working

A common issue raised by a number of respondents concerns the way that VCS organisations are funded [45]. These broad areas of concern were based around the fact the organisations have to vie for different pots of money to provide specific services for the particular remit of client group.

A similar issue was raised by one respondent who suggested that there may be a fear of competition amongst partner organisations culminating in the concern that -

*they are going to steal my clients. What service am I going to be left to run? *(R4)

There was also a concern that excessive attention is given to those things that are easier to measure at the expense of those things that were harder to quantify.

Furthermore, some 'causes' are perceived as more worthy resulting in larger funding pools for certain projects. One respondent describes the inequity in accessing project funding for lesbians:

If you work in BME communities, or people with HIV, it can be a lot easier to get funding.(R4)

Despite a good deal of praise for the practice of partnership working (see below) one respondent drew attention to its potentially problematic nature, stating that co-working or partnership working may be perceived as synonymous with "extra work" and therefore maybe avoided.

#### Attitudes and constraints of the statutory services towards the VCS

Three of the respondents were explicit in their concerns around the relationship between themselves and statutory sector organisations.

One expressed concern about the attitude held by the statutory sector towards the VCS. He suggested that the use of the term 'third sector' with reference to the VCS reinforces a view that this sector is somehow of less value:

*the image the statutory sector has of the voluntary sector is 'there, there nice little organisations. You do your specialist thing and leave us to do the professional work*. (R3)

One respondent made explicit their frustrations with the statutory sector suggesting that social services are not equipped to deal with the complexity of problems faced by some young people:

*if that young person doesn't co-operate, for whatever reason, then they're [social services] not interested and the young person is left high and dry*. (R7)

Problems of access are also faced by the VCS themselves:

*as an agency, it's also difficult to access these services on behalf of young people, especially when the average user will have more than one, like sort of, special need*. (R7)

#### Expectations/limitations of services

Expectations or preconceptions of the VCS services may also become a barrier in terms of accessing 'hard to reach' groups. This is encapsulated by one respondent, who states:

*it is about ourselves, the expectations that you must come to us*.(R5)

It is suggested that for a service that is dominated by clients from a more narrow demographic this can become a barrier for potential service users who may feel like "outsiders" (R3). Even within services for the 'hard to reach' there is a hard core of individuals who do not access services. In some homeless day centres, for example, where the client group are predominantly white men, then women and people from other ethnic groups may feel more reluctant to utilise the service.

Furthermore, the views held by some clients about other service users can contribute to the difficulties faced by service providers in serving diverse groups:

*we see individuals coming in with a very blinkered view about some of the service users that we see, maybe refugees - "why are they over here stealing our jobs? *(R4)

### Facilitators

As described, there are a number of perceived barriers in terms of providing services to the 'hard to reach.' Conversely, a number of facilitators in providing accessible services to the difficult to engage were cited.

#### Treatment of Clients - trust, respect

A perception that service users have been poorly treated by other services, and in particular by statutory service has already been raised.

The most commonly cited facilitator for engaging clients was the way VCS services communicate with and treat clients.

This key theme is illustrated by the following quotations from R1:

*we don't interrogate clients when they come here. We ask what questions we need to give them the service they want*. (R1)

Reference is also made to the attention paid to the attitude of the staff members:

*I think the important thing is to spend an awful lot of time and effort on making sure that staff are genuinely welcoming and non-judgmental about absolutely everyone that walks through the door*. (R1)

The quality of the relationship that develops between staff and service user is a major factor in engaging clients; building trust and respect; being non-judgmental and being able to relate to and empower people.

Ok, you can trust us, but it is very much about them being able to recognise that they can trust us, or build up that relationship which can take weeks. (R3)

#### Flexibility

Offering flexible services that respond to the needs of the service users including running outreach services, listening to feedback, offering flexible opening hours and providing service users with the kinds of services they want were all key facilitators cited by the respondents.

One respondent made the point that although they are not as "flexible as we would like," many of the people using this service are

*very much on the peripheries of normal society... a lot of people on benefits ...so they can come as and when*. (R4)

Furthermore, by offering an outreach service to encourage potential service users to engage, they are able to access people who may not ordinarily feel comfortable to attending a HIV service. Not all the organisations interviewed conducted outreach and this was largely due to resource limitations. There appeared to be a tension between offering a service to all the target audience, including those 'harder to reach' clients and being able to provide an adequate service to all.

Offering services that people want is a key facilitator for engagement. One respondent described how changing their opening times as well as offering service users a hot meal had resulted in a fifty per cent increase in demand for the service (R3).

#### Partnership Working

As described above there are a number of drawbacks to partnership working that can be perceived as barriers to service participation. However, there are also a number of positive aspects to working in partnership and it seemed to be a priority for a number of the organisations.

One respondent makes the point that working in partnership is actively encouraged by the funding bodies who support organisations.

Partnerships can be useful in assisting in:

*getting information out there, and ensuring that people know who we are*. (R4)

Acknowledgement of the limitations of a service was also important:

*we don't claim to know everything. We don't want to do everything. It isn't possible to do everything really, really well, but what you can do is build very, very strong partnerships with other organisations that do specialise in particular areas*. (R5)

#### User involvement

For some of the respondents, emphasis was placed upon the importance of user participation. Service participation and interview panels were conducted at one organisation throughout the year. "Member helpers" or trainee volunteers form part of the team with the aim to build the esteem and skills of the service users. Using service users as part of the team encourages other clients to participate and feel more settled:

*It's about having a different relationship. A service user can better describe it [how it feels to be a service user]. They can share that with each other, use that as a bridging gap and a way of opening doors*. (R3)

The kinds of relationships the VCS is able to develop with their clients is of the kind that is often found wanting in statutory services. The emphasis they place on user involvement and use of volunteers as demonstrated above can act as an enabler for those they seek to serve.

## Discussion

### The VCS a model for the NHS?

As we have seen, tackling health inequalities is a top priority for the Government and part of this includes engaging those 'seldom seen, seldom heard' in services.

Recent Department of Health initiatives have highlighted the need for improved service provision and configuration. The NHS Next Stage Review Interim Report [46] reported that despite 'sustained investment and improvement in the NHS over the past 10 years, access to primary medical care and services and quality of those services continues to vary significantly across the country.' (pp 3)

As noted by a number of the respondents, the importance of listening to the voices of users has been taken onboard by the NHS as a key resource in providing appropriate services [47]. Already, changes in how health services, and in particular primary care services, are delivered seem to bear the hallmarks of VCS practice, but there is a need to go further. Some of the changes in provision of GP services aimed at improving patient access and experience include the introduction of patient feedback and quality assessment (surveying patients about their local GP practice); the extension of opening hours to accommodate the varying lifestyles of patient populations and thus engaging with the 'seldom seen'. As we have seen the VCS ability to offer flexibility in terms of opening hours and responsive services for service users are cited as facilitators for the more difficult to engage.

The Department of Health acknowledges the need to 'establish effective links with frontline services, utilising the potential of VCF sector agencies as valuable catalysts for dialogue, mutual understanding and empowerment [48] (pp4).

Partnership working is becoming increasingly important to the health service.

From the perspective of primary care provision it is acknowledged that as commissioners of services, Primary Care Trusts (PCTs) working alongside local authorities should 'identify specific communities with particularly poor health, such as travellers, migrant workers, people with learning disabilities, those living in disadvantaged areas or demographic groups' [49].

It seems that the NHS is moving in the right direction in terms of offering choice and a voice to patients. Before equity in health services can be fully realised that work must continue in engaging the most excluded and 'hard to reach' in society.

### Study Limitations

The study is clearly limited by the small sample size. Most qualitative studies will aim to interview between twenty and thirty people although the aim is to continue data collection until data saturation is met. This is the point were no further themes are being generated. Unfortunately, only eight out of the thirty organisations felt they had the time to participate. It could be postulated that organisations providing services for more marginal groups are resource and time limited and therefore find it more difficult to put time aside, so the views of practitioners from these organisations may have been lost. The participant organisations operate services in a large, diversely populated urban area, and the kind of experiences and views found here are likely to differ from those from organisations operating in rural communities, with a widely dispersed population. There are also likely to regional variations that are not reflected in this study.

However as a pilot study, one of the objectives is to provide background to inform a larger scale study proposal which aims to involve the views of a larger number of participants including service users and primary care practitioners.

## Conclusion

The VCS have a long history of working with marginalised, disadvantaged and 'hard to reach' groups and as discussed their experiences may provide potential lessons for statutory service providers, particularly health services.

The VCS is able to provide added value in terms of the resources they offer; the procedures they employ and how they are organised [30].

From the interviews conducted there are four main areas of importance that have arisen that relate to how best to engage 'hard to reach groups': attitude of staff; service flexibility; working in partnership with other organisations and empowering users involvement. In order to fully engage with the 'hard to reach' and provide an equitable health service for all, the NHS must embrace some of the philosophies that appear to underpin the VCS.

## Competing interests

The authors declare that they have no competing interests.

## Authors' contributions

SF conducted the interviews and drafted the original manuscript. BH participated in its design and coordination and helped to draft the manuscript. All authors read and approved the final manuscript.

## Pre-publication history

The pre-publication history for this paper can be accessed here:

http://www.biomedcentral.com/1472-6963/10/92/prepub
